# A rare multiple myeloma complication as a spinal cord metastasis: A case report

**DOI:** 10.1002/ccr3.7069

**Published:** 2023-03-08

**Authors:** Marah Mansour, Zeinab Haider, Lin Al Khiami, Mohammad Saleem Talal Sioufi, Asmaa Takieddin, Aiman Abo Al Shamat, Ricarda Alwaw, Mais Musleh

**Affiliations:** ^1^ Faculty of Medicine Tartous University Tartous Syria; ^2^ Faculty of Medicine Al andalus University for Medical Sciences Qadmus Syria; ^3^ Faculty of Medicine Damascus University Damascus Syria; ^4^ Department of Neurology Al Assad University Hospital Damascus Syria; ^5^ Department of Hematology Al Assad University Hospital Damascus Syria

**Keywords:** case report, chemotherapy, CNS, complications, malignancy, multiple myeloma

## Abstract

Multiple myeloma is a hematological cancer mostly located in the marrow of the vertebrae, pelvis, and thighs. Although the presence of extramedullary disease in the central nervous system is rare, herein, we report a complicated case of multiple myeloma in the spinal cord.

## Graphical abstract

Multiple myeloma is a malignant genetic disease that mostly affects the bone marrow. Spinal cord metastases are an uncommon complication. Surgical decompression should be performed early to avoid deterioration.

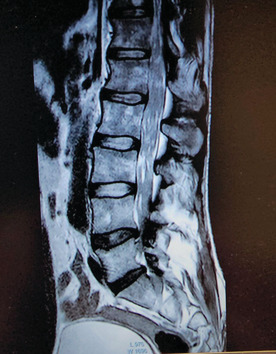

## 1 BACKGROUND

Multiple myeloma (MM) is hematological cancer characterized by the growth of malignant plasma cells of B‐cell lineage which are found in the bone marrow and secrete immunoglobulins (Ig),^1^, ^2^, ^3^ The illness mostly affects the bones, although it can also affect the lymphatic nodes and epidermis. MM is an incurable disease,^3^ which is also one of the most prevalent cancers that affect the spine.^4^ In France, approximately 5000 new cases are detected each year,^2^ while in the United States, MM represents around 1.6% of all malignant tumors and almost 10% of hematologic malignancies.^1^, ^5^, ^6^ Males are somewhat more likely than females to have MM and African‐Americans are twice as likely as Caucasians to develop the disease, The patient's median age at the time of discovery is around 65 years old.^1^, ^6^ Some of the most common clinical signs (seen in 80% of patients) are refractory pain, fracture, vertebral collapse, or spinal cord compression due to bone lysis. Anemia, hypercalcemia, renal dysfunction, recurring infections, and hyperviscosity are some symptoms of its progression.^2^, ^6^, ^7^ One of the pathognomonic and diagnostic hallmarks of MM is bone injury, which appears as an osteolytic bone disease (OBD) or osteopenia,^7^ OBD affects approximately 90% of patients with MM.^7^, ^8^ Because of that injury in MM patients, imaging plays an important role in disease management. Magnetic resonance imaging (MRI) has proven to be a useful tool in the diagnosis of MM bone lesions.^8^ Clinical, radiographic, histopathologic, and laboratory findings are frequently used to diagnose MM. Back pain, vertebral fractures, paresthesia, and paresis owing to spinal cord compression are the most common symptoms of axial skeleton metastases in symptomatic patients. Hypercalcemia can develop from lytic bone lesions, and renal failure might manifest as anemia and proteinuria. The International Myeloma Working Group (IMWG) established calcium elevation, renal dysfunction, anemia, and bone disease (CRAB) acronym to describe the clinical symptoms of MM and to distinguish it from other plasma cell dyscrasias such as solitary plasmacytoma.^5^ For a long time, prednisone, melphalan, and other corticosteroids were used to treat MM,^9^, ^10^ Proteasome inhibitor medications like Velcade (bortezomib), in conjunction with dexamethasone (the VD regimen), have been utilized to treat MM for the past 20 years. Velcade (bortezomib) and dexamethasone were typically combined with cyclophosphamide, adriamycin, or even thalidomide (VTD) to increase efficacy.^9^, ^10^ In this study, we report a case of MM which was complicated with spinal cord metastasis.

## 2 CASE PRESENTATION

**FIGURE 1:**
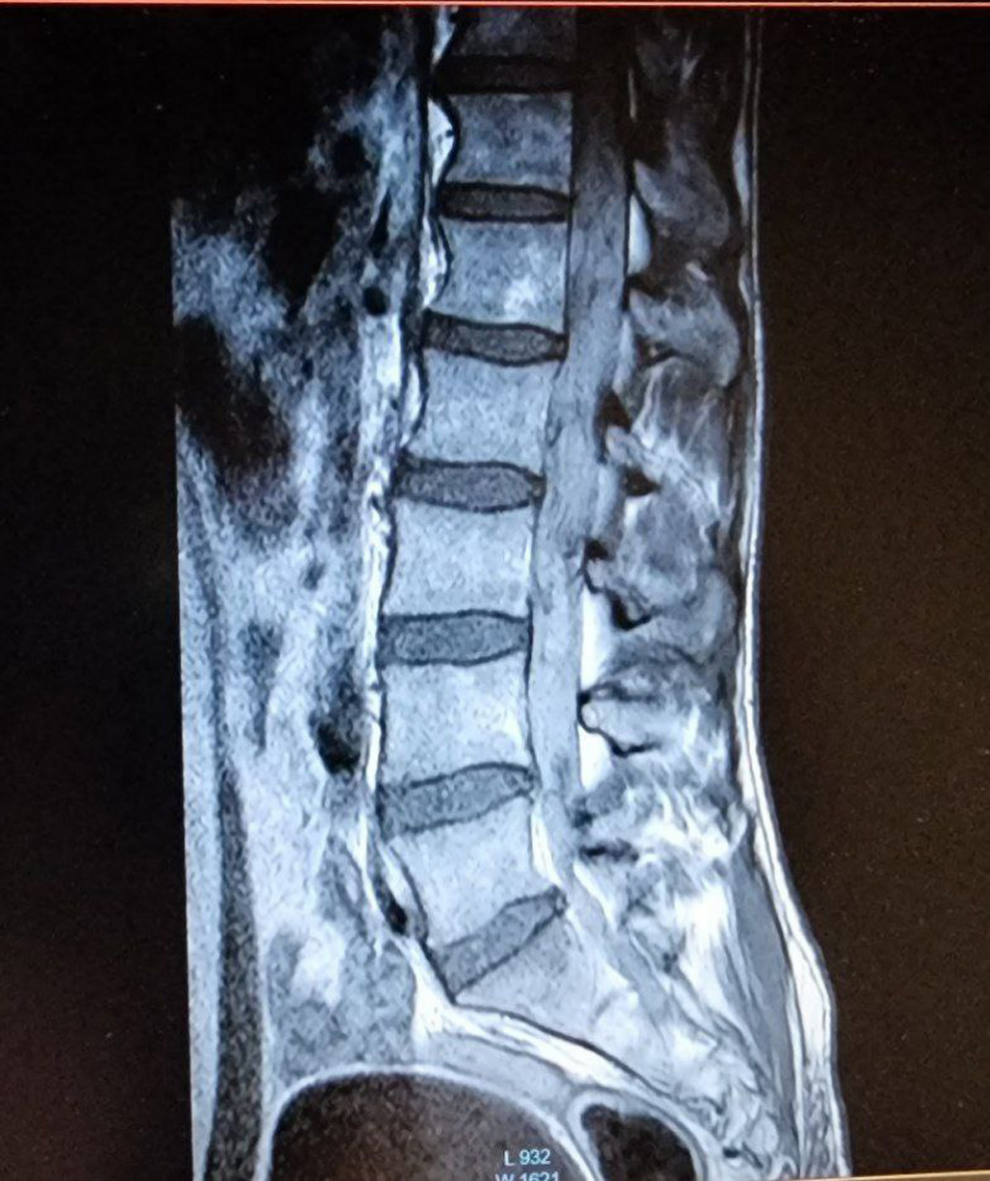
MRI of the lesion in the dorsal and lumbar columns of the spinal cord (T1).

**FIGURE 2:**
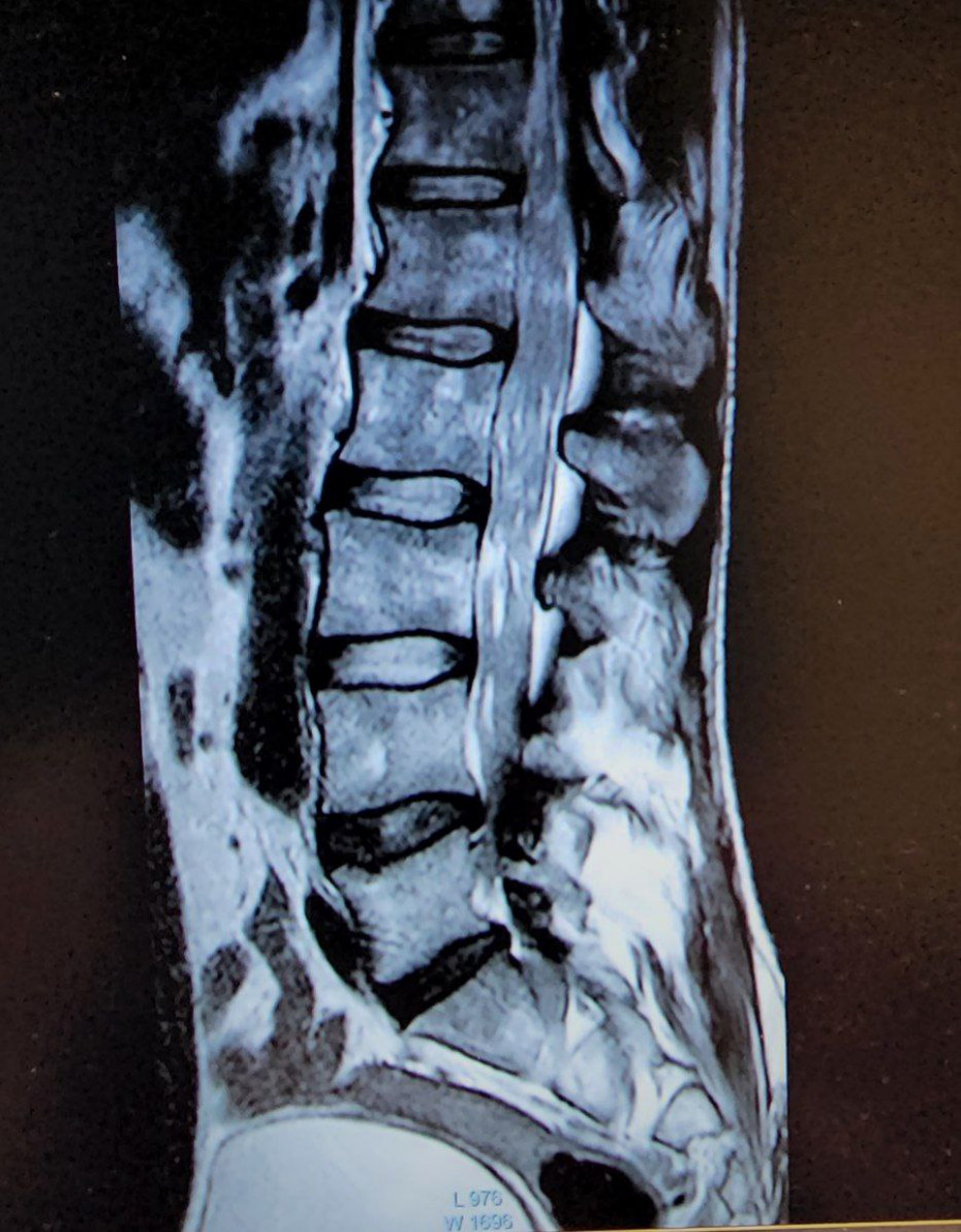
MRI of the lesion in the dorsal and lumbar columns of the spinal cord (T2).

**FIGURE 3:**
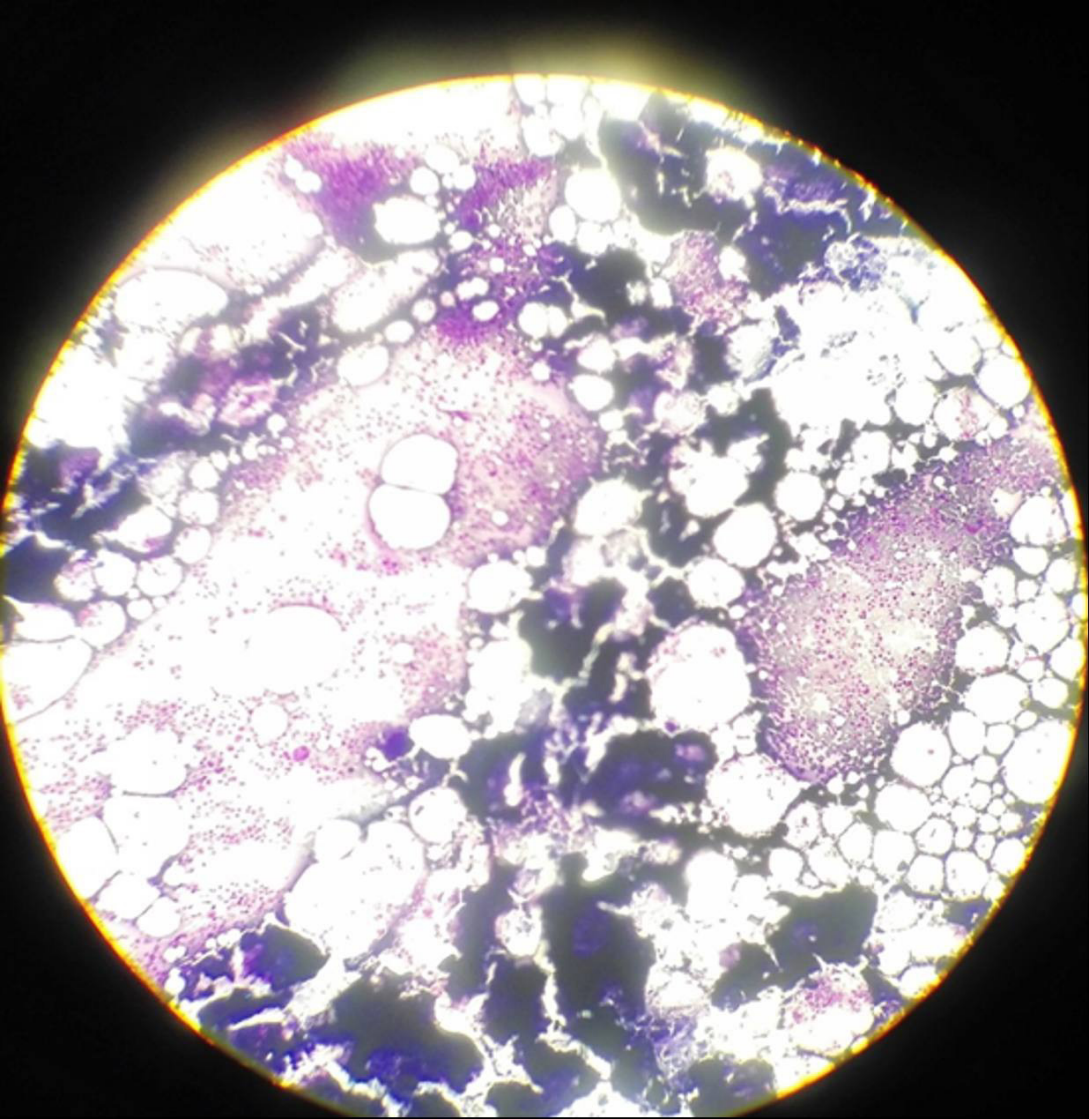
Myelo Centesis on an optical microscope Giemsa staining, magnification 10, showing good purified cells.

**FIGURE 4:**
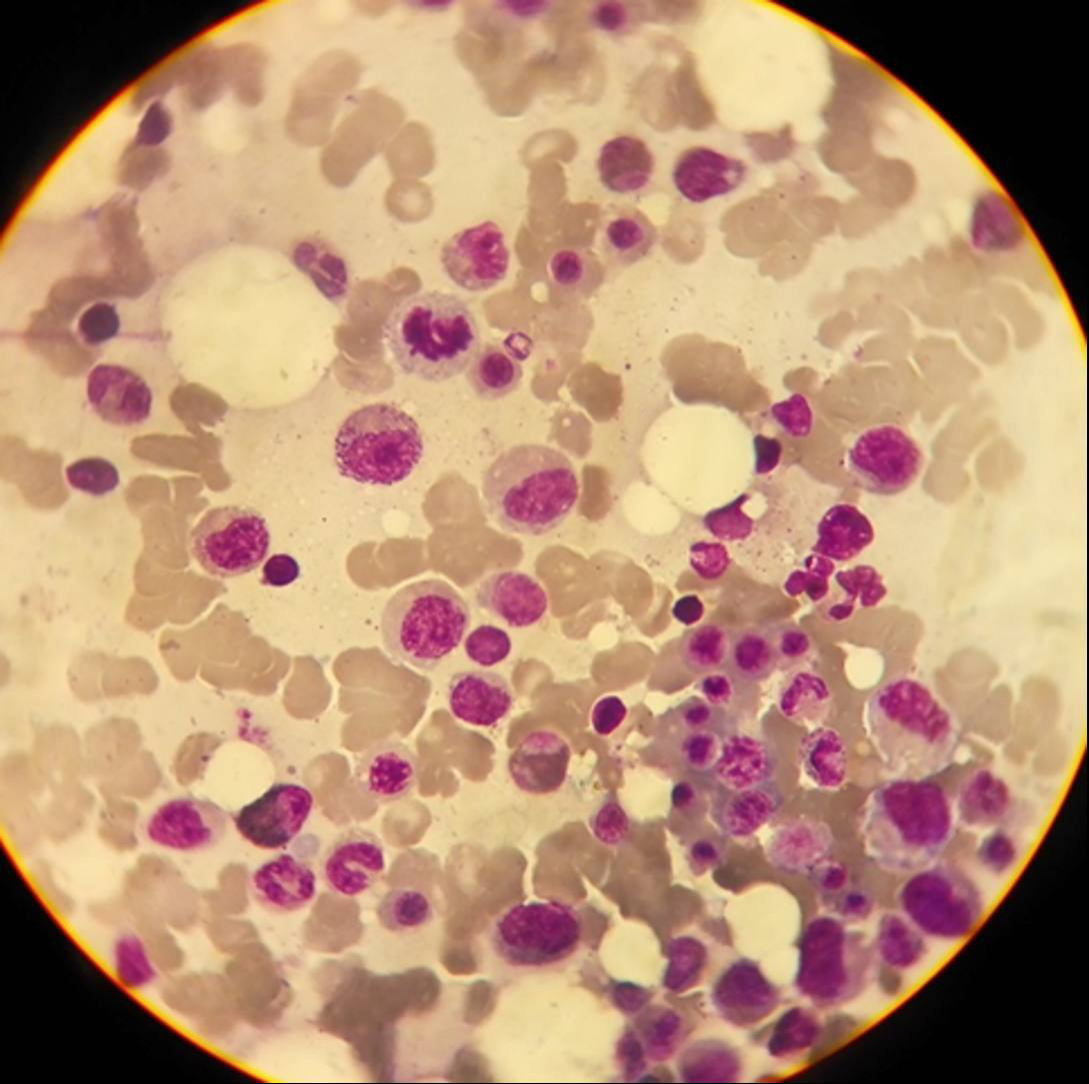
Myelo Centesis magnified 100 Giemsa staining showing marrow cytology and plasma infiltration in the bone marrow.

**FIGURE 5:**
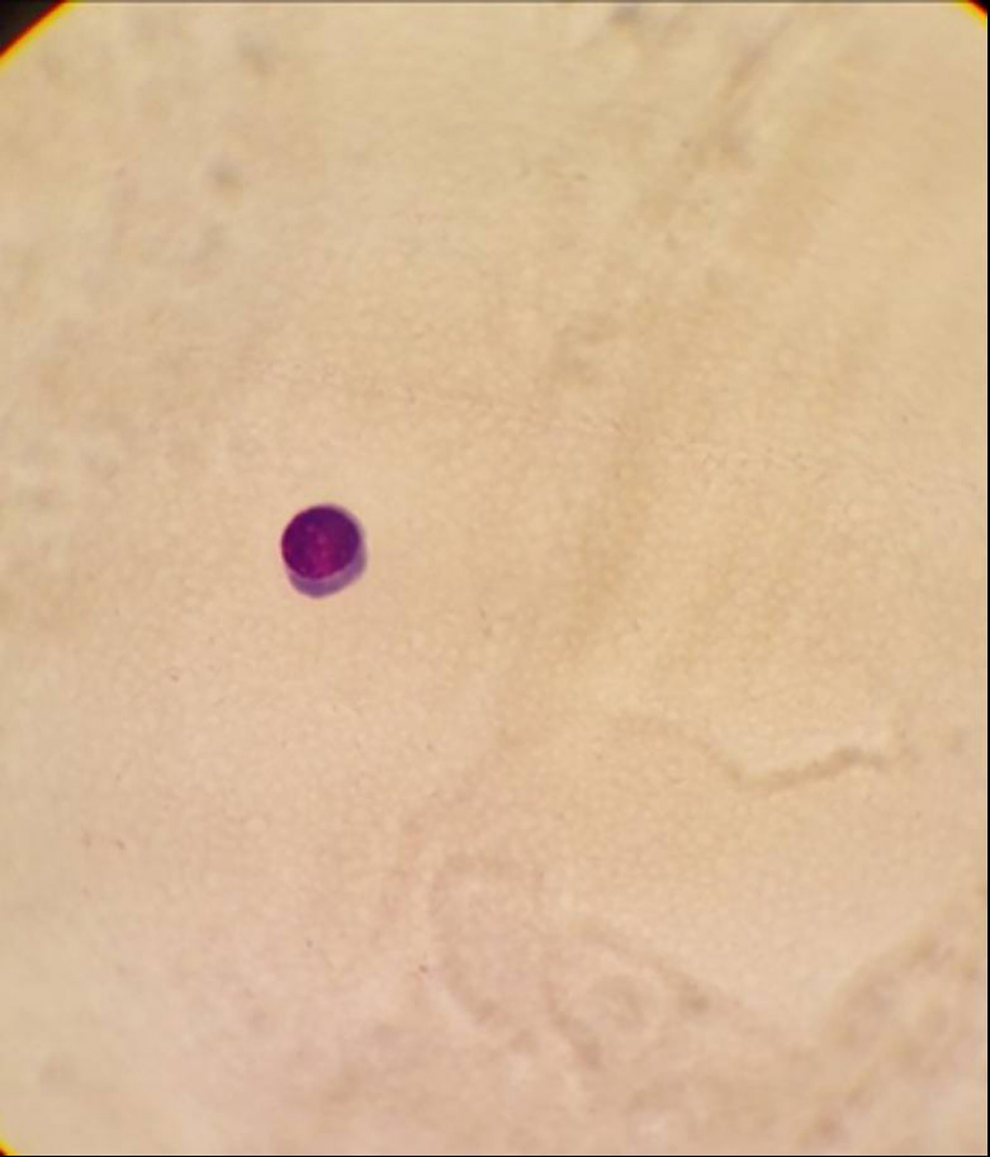
Magnification of CSF puncture 100 Giemsa staining showing atypical plasma cells infused with CSF.

A 41‐year‐old male presented to the Department of Neurology with a complaint of weakness in the lower extremities with a ⅖ strength, uncontrolled sphincters, and a lack of reflexes. The patient was diagnosed with MM 10 months ago and had myeloid infiltration with plasma cells of more than 60% at the expense of Immunoglobulin G (IgG) Kappa. The patient has received four rounds of Velcade® (bortezomib) 1.3 mg/m2 (milligram per square meter of body mass) on days 1,4, 8,11, and 22 + Revlimid® (lenalidomide) 25 mg from day 1 until day 21 + dexamethasone 40 mg for 4 days [VRD] treatment, followed by an autologous transplant, and the patient was in remission. The patient's vital signs and laboratory tests were normal, and the MRI of the dorsal and lumbar columns showed a mass at the end of the spinal cord extending to infiltrate the entire cauda equina from the level of the second dorsal vertebra to the sacrum with a high suspicion of astrocytoma (Figures 1 and 2). Cerebrospinal fluid (CSF) puncture showed that the patient had atypical plasmatic infiltrates (Figure 3). The immunophenotype of the CSF revealed that 38% of the cells were plasma cells, with a positive cluster of differentiation 38 (CD38), syndecan‐1 (CD138), ig kappa, and cluster of differentiation 7 (CD7) cells in the fluid noting that the patient was in remission; the bone marrow aspiration results illustrated the existence of less than 5% of plasma cells and no monoclonal peak was found in the peripheral blood sample (Figures 4 and 5). To prepare for emergency irradiation, intrathecal methotrexate (IT MTX) was given five times with a high dose of Dixon. Nevertheless, the patient passed away directly after; therefore, we were unable to track the patient's progress in this case.

## 3 DISCUSSION

MM is a hematological malignant neoplasm defined by abnormal proliferation of B‐cell lineage malignant plasma cells in the bone marrow and the release of a significant number of monoclonal immunoglobulins.^3^, ^11^ MM accounts for 1% of all cancers and 10% of all hematologic malignancies. In the United States, about 32,000 new cases are diagnosed each year, with nearly 13,000 patients dying from the condition, and the yearly age‐adjusted incidence has remained stable at around four per 100,000 for decades.^6^ Geographically, Australia, Europe, and North America have the greatest incidences of the disease.^3^ Males are more likely than females to develop MM, and African‐Americans are twice as likely as Caucasians to develop the disease. Patients' median age at the time of diagnosis is around 65 years old.^6^ The marrow‐containing bones of the vertebrae, pelvis, and femur are the most prevalent sites for MM, but lesions can occur in any bone.^3^ The majority of tumor cells are restricted to the bone marrow. However, malignant plasma cells can break through the bone marrow and periosteal tissue, creating tumorous masses in the nearby bone site. Additionally, the cells may enter the bloodstream and invade the distal tissue to produce tumorous masses known as extramedullary multiple myeloma (EMM). Extramedullary bone–related (EMB) refers to the adjacent bone, while extramedullary extraosseous (EME) refers to the extramedullary plasmacytoma distal to the bone.EMM can be found in the diagnosis of MM or the recurrence of the disease, with an incidence of 3–30% in the diagnosis and 6%–40% in the refractory relapse of the disease. EMB has an incidence rate of 6%–35%, while EME has an incidence rate of 0.5%–3.5%.^11^ In our case, a 41‐year‐old male diagnosed with MM in the vertebrae, a spinal cord metastasis was revealed as a recurrence of the disease. EMM, which occurs outside of the bone marrow, has a poor prognosis and can rarely infiltrate the central nervous system (CNS), with about 1%–2% of patients with MM developing a secondary CNS malignancy, resulting in neurologic deficits, disability, and a lower quality of life. The most common form of CNS EMM is intracranial metastasis, which is thought to be caused by hematogenous dissemination or contiguous seeding from local lytic bone lesions. Intramedullary spinal cord metastases, on the other hand, are extremely uncommon. In the last 17 years, seven cases with intramedullary spinal cord MM or plasmacytoma metastasis have been recorded, including our patient. Six of the patients were males and one was a female patient. From the cervical cord to the cauda equina, the level of spinal cord metastases varied.^5^ Although our patient is the first to present with a metastasis extending from the level of the second dorsal vertebra (T2) to the entire cauda equina. MM has an etiology that is unknown. Occupational/environmental risk factors such as asbestos, petroleum, farming, and ionizing radiation have been confirmed to have an association with the disease in some studies. MM is caused by multiple genetic mutations in plasma cells and the immunoglobulins they produce, although some cases of familial MM, which suggest a hereditary basis, have been reported. MM tumors can develop spontaneously or as a result of the pre‐malignant condition known as “monoclonal gammopathy of unknown significance” (MGUS).^3^ MGUS affects more than 3% of the population over the age of 50. At a rate of 1% each year, it develops into MM or related cancer.^6^ The mechanisms that lead to the progression of MGUS to myeloma are uncertain. However, they share gene translocations that code for both heavy and light chains (IgH and IgL). Abnormalities on chromosome 13 are also common, occurring in around half of all cases, and are linked to a worse prognosis.^3^ MM has a wide range of clinical manifestations. The key concerns are referred to as CRAB (hypercalcemia, renal impairment, anemia, and bone lesions). The presence of a combination of these symptoms should raise the diagnostic suspicion of myeloma.^12^, ^13^ However, muscle weakness is the most common presenting neurologic deficit in patients with spinal cord metastasis, occurring in five of seven cases reported in the literature, including our patient, who presented with weakness in the lower extremities with a ⅖ strength, followed by paresthesia in two of seven cases, and loss of tendon reflexes in two cases, including our case. Paresis, paraparesis, tetraparesis, sensory deficit, ataxia, and gait difficulty are among the other symptoms.^5^ Our patient also had hypotonia and uncontrolled sphincters. This disease's differential diagnosis includes MGUS, plasmacytoma, astrocytoma, and lymphoma.^5^, ^13^ The IMWG has devised diagnostic criteria to effectively distinguish between them and MM.^12^ The presence of monoclonal protein in the serum or urine, with a positive bone marrow biopsy revealing more than 10% plasma mono‐clonal cells and at least 1 finding for organ failure according to the CRAB acronym, is indicative of MM.^12^, ^13^ In our case, the bone marrow biopsy showed more than 60% IGG kappa plasma cell infiltration. After treatment and during the new onset of symptoms, a second biopsy was performed and displayed less than 5% infiltration and no monoclonal protein peak in the blood smear electrophoresis, which reflects a state of inactivity of the disease in the bone marrow (Figure 3). MM affects the bone, especially the axial skeleton, in up to 90% of patients.^7^ Bone lytic lesions were previously diagnosed using a conventional skeletal survey. But there is a deficiency in the sensitivity it offers, so it should only be used when other modalities are unavailable. Whole body low dose has a higher sensitivity in showing lytic bone lesions, where it reflects signs of bone loss, especially in the spine and the pelvis.^14^ On the other hand, MRI has been established as the imaging modality of choice when diagnosing MM by numerous studies.^3^, ^4^, ^5^, ^13^ MRI can show cell infiltration in the bone marrow which could indicate the presence of MM. The IMWG recently updated the MRI indication for diagnosing MM by increasing the number of focal lesions present on the image that are greater than 5 mm.^14^ The MRI sequence for our case showed a mass at the bottom of the spinal cord invading the cauda equina, extending from the second dorsal vertebra all the way to the sacrum (Figures 1 and 2). After MRI, to avoid postlumbar puncture image changes, CSF analysis using lumbar puncture would be beneficial in confirming meningeal involvement by detecting malignant plasma cells.^13^ In our case, CSF analysis showed atypical plasma cells. Furthermore, we performed immunophenotyping of the CSF sample, which revealed CD38, CD138, IGG kappa, and CD7 (Figures 4 and 5). There are multiple and different approaches to treatment for MM. But since there have not been a lot of CNS‐involved MM cases, the treatment still does not have regulated guidelines. Therapy for these cases requires acting agents on the MM and the ability to penetrate the blood–brain barrier.^5^ The different treatment plans for MM without CNS metastasis include chemotherapy such as bortezomib, thalidomide, lenalidomide, and bendamustine; radiotherapy, which is ideal for the plasma cell neoplasms are radiosensitive; and autologous stem cell transplant (ASCT).^5^, ^6^, ^7^, ^10^, ^13^ Bisphosphonates are pyrophosphate equivalents that are used as osteoclast inhibiting agents.^14^ They also have a proliferating effect on the osteoblasts and osteocytes hindering cell death.^7^ These changes result in fewer skeletal‐related events such as pathological fractures and spinal cord compression.^3^ However, bisphosphonates could induce renal function impairment and osteonecrosis of the jaw.^3^, ^7^ Bortezomib, which is a proteasome inhibitor, limits osteoclastogenesis and helps to regulate the bone remodeling feature of MM.^6^ It is usually used alongside dexamethasone as a part of the VD protocol.^10^, ^11^ Immunomodulating agents such as Thalidomide and Lenalidomide could be added to the protocol where they will be named VTD or VRD respectively.^10^ Our patient received four courses of VRD, followed by complete remission of the disease.

ASCTs have shown great promise in increasing progression‐free survival and overall survival rate. A recent retrospective study, conducted on 80 patients with EMM, where 13 patients received ASCT, showed higher survival rates for patients receiving ASCT. The progression‐free survival rate was 46, and the overall survival rate was remarkably greater than rates in patients who have not had ASCT.^11^ In this case, following chemotherapy, there was little to no trace of the neoplastic cells in the bone marrow.

## 4 CONCLUSION

MM is a multifocal neoplastic genetic disease that mainly affects the bone marrow.

Spinal cord metastasis is a rare complication which is associated with a poor prognosis.

Surgical decompression should be considered early in management to relieve symptoms and prevent further deterioration.

Further research is needed to develop new protocols in order to improve overall survival rates.

## AUTHOR CONTRIBUTIONS

**Marah Mansour:** Conceptualization; data curation; formal analysis; investigation; methodology; project administration; resources; software; supervision; validation; visualization; writing – original draft; writing – review and editing. **Zeinab Haider:** Conceptualization; data curation; formal analysis; investigation; methodology; resources; software; visualization; writing – original draft; writing – review and editing. **Lin Al Khiami:** Conceptualization; data curation; formal analysis; investigation; methodology; resources; visualization; writing – original draft; writing – review and editing. **Mohammad Saleem Talal Sioufi:** Conceptualization; data curation; formal analysis; investigation; methodology; software; visualization; writing – original draft; writing – review and editing. **Asmaa Takieddin:** Conceptualization; data curation; formal analysis; investigation; writing – original draft; writing – review and editing. **Aiman Abo Al Shamat:** Conceptualization; data curation; formal analysis; investigation; writing – original draft; writing – review and editing. **Ricarda Alwaw:** Conceptualization; data curation; investigation; methodology; project administration; resources; supervision; writing – original draft; writing – review and editing. **Mais Musleh:** Conceptualization; data curation; formal analysis; investigation; writing – original draft; writing – review and editing.

## FUNDING INFORMATION

No funding was required.

## CONFLICT OF INTEREST STATEMENT

The authors declare that they have no conflicts of interest.

## ETHICAL APPROVAL AND CONSENT TO PARTICIPATE

Not required for this case report.

## CONSENT

Written informed consent was obtained from the patient for publishing this case report and any accompanying images. A copy of the written consent is available for review by the Editor‐in‐Chief of this journal on request.

## GUARANTOR

Mais Musleh is the guarantor of this work.

## Data Availability

Not applicable. All data (of the patient) generated during this study are included in this published article and its supplementary information files.
